# Utility of Marine Benthic Associations as a Multivariate Proxy of Paleobathymetry: A Direct Test from Recent Coastal Ecosystems of North Carolina

**DOI:** 10.1371/journal.pone.0095711

**Published:** 2014-04-21

**Authors:** Carrie L. Tyler, Michał Kowalewski

**Affiliations:** Florida Museum of Natural History, University of Florida, Gainesville, Florida, United States of America; University of Tasmania, Australia

## Abstract

Benthic marine fossil associations have been used in paleontological studies as multivariate environmental proxies, with particular focus on their utility as water depth estimators. To test this approach directly, we evaluated modern marine invertebrate communities along an onshore-offshore gradient to determine the relationship between community composition and bathymetry, compare the performance of various ordination techniques, and assess whether restricting community datasets to preservable taxa (a proxy for paleontological data) and finer spatial scales diminishes the applicability of multivariate community data as an environmental proxy. Different indirect (unconstrained) ordination techniques (PCoA, CA, DCA, and NMDS) yielded consistent outcomes: locality Axis 1 scores correlated with actual locality depths, and taxon Axis 1 scores correlated with actual preferred taxon depths, indicating that changes in faunal associations primarily reflect bathymetry, or its environmental correlatives. For datasets restricted to taxa with preservable hard parts, heavily biomineralized mollusks, open ocean habitats, and a single onshore-offshore gradient, the significant correlation between water depth and Axis 1 was still observed. However, for these restricted datasets, the correlation between Axis 1 and bathymetry was reduced and, in most cases, notably weaker than estimates produced by subsampling models. Consistent with multiple paleontological studies, the direct tests carried out here for a modern habitat using known bathymetry suggests that multivariate proxies derived from marine benthic associations may serve as a viable proxy of water depth. The general applicability of multivariate paleocommunity data as an indirect proxy of bathymetry is dependent on habitat type, intrinsic ecological characteristics of dominant faunas, taxonomic scope, and spatial and temporal scales of analysis, highlighting the need for continued testing in present-day depositional settings.

## Introduction

Relative species abundance can frequently be described as a function of measured environmental variables (direct gradient analysis), as community composition varies along environmental gradients. Conversely, environmental gradients may be inferred by detecting patterns of variation in community composition (indirect gradient analysis) [1]. The latter approach is frequently employed in paleoecological analyses (e.g., [2], [3], [4]), and consists of arranging community samples along axes of variation based on their composition, followed by interpretation of the axes in terms of environmental gradients [5]. Indirect gradient analysis is typically performed using multivariate ordination techniques applied to community abundance data [1]. These techniques allow for plotting samples in ordination space, to capture the major directions of variation in faunal composition. Ordination axes are then interpreted *post-hoc* in terms of putative eco-environmental gradients controlling species composition and sample distribution. Present-day settings allow for a direct comparison of ordination scores derived from community data against actual environmental variables. This study employs modern benthic invertebrate communities to directly test the reliability of depth estimates derived from indirect ordinations of quantitative community data.

In present-day ecosystems changes in environmental conditions associated with water depth, such as decrease in light intensity, decrease in wave energy, changes in ambient temperature, and changes in salinity (related to distance from shore and precipitation), are often reflected by fundamental differences in taxonomic and ecological composition of marine benthic communities [6], [7], [8], [9], [10], [11]. In the fossil record faunal composition is also likely to change notably with water depth (e.g., [12], [13], [14], [15], [16]), a view often supported by indirect multivariate analyses of fossil associations evaluated against independent lithological and/or ecological proxies (e.g., [4], [13], [17], [18], [19], [20], [21], [22], [23]). Many modern ecological studies, however, identify factors other than depth as primary controls (e.g., [24], [25], [26], [27], [28]). Furthermore, most paleontological studies are limited to one, or at most a few, higher taxa (but see [13], [23], [29]), particularly heavily biomineralized organisms such as brachiopods and mollusks (e.g., [4], [22], [30]). Because studies examining the effect of preservation biases on community ordination patterns are lacking, it remains unclear whether ordinations based on subsets of communities, confined to biomineralized organisms, accurately detect ecological gradients observed for the entire community.

In this study, dredge samples collected along an onshore-offshore gradient off the coast of North Carolina, were used to assess the reliability and fidelity of multivariate community proxies of bathymetry. Using the resulting benthic invertebrate community data, we evaluated four research questions:

First, we assessed the hypothesis that depth estimates based on faunal composition provided reliable measures of actual bathymetry (and related environmental parameters). Under this hypothesis, indirect ordination scores derived solely from faunal composition data are expected to correlate with locality water depth (known *a priori*, and independent of faunal data). This hypothesis predicts that indirect ordination scores of species should correlate with the actual preferred species depth, which can be estimated directly in modern settings via direct ordination methods (weighted averaging *sensu* [31]).

Second, by deriving ordinations using various subsets of the community, we assess the multivariate fidelity of the marine benthic associations from a paleontological perspective; specifically, comparing the performance of indirect ordinations for all taxa, preservable taxa, and heavily biomineralized mollusks. These three datasets, which represent neontological data (all taxa) and two paleontological proxies (preservable taxa and heavily biomineralized mollusks), respectively, are used here to evaluate their mutual consistency and compare their relative effectiveness in capturing environmental information.

Third, we compare the results of Principal Coordinates Analysis, Correspondence Analysis, Detrended Correspondence Analysis, and Non-Metric Multidimensional Scaling, in terms of their consistency and their effectiveness in deriving robust environmental proxies from multivariate community datasets.

Finally, we examine how geographic scope of the analysis alters the strength of the bathymetric signal, as the relationship between community composition and depth is expected to scale spatially and temporally [10], [20], [32]. Results are compared for the entire study area (∼952 km^2^), open ocean habitats (∼578 km^2^), and a confined onshore-offshore gradient (∼180 km^2^).

### Study Area Description

The coast of North Carolina is protected from the open ocean by barrier islands and sandbars. Storm activity is concentrated in the fall and winter (September-February), with waves arriving predominantly from the northeast during winter months and from the southeast during summer months. The area behind these islands and bars, referred to as the back-sound, is typically more lagoonal/estuarine and somewhat sheltered from swells and storms. The average salinity of the open marine waters in the region is 36 ppt, with the warm Gulf Stream flowing from the south. Inner shelf waters vary seasonally in temperature (>28°C in summer, 12–14°C in winter) [33]. Nearshore average salinity is 34 ppt, and estuarine waters vary in salinity levels dependent on precipitation. Water depth is relatively shallow on the continental shelf, and increases gradually to ∼70 m with increasing distance from shore to the edge of the continental shelf [33]. The shelf break (∼120 km off the coast of Onslow Bay) marks a sudden dramatic increase in depth. Sediments include fine and medium to coarse sands and gravel [33].

### Ethics Statement

All necessary permits were obtained for the described study, which complied with all relevant regulations. No permits are required for general dredging in the study area. As all dredging was conducted using the Duke University Marine Lab (DUML) facilities and equipment, field collections fell under the DUML invertebrate collections permits (Duke University Marine Lab Scientific or Education Permit 707075 for 2011, 2012, and 2013). With the exception of species identification voucher specimens, all individuals were released *in situ* after counting and identification. No protected species were identified in the sampled material. Data and R script to perform all analyses are included in supplemental materials.

## Materials and Methods

**Figure 1:**
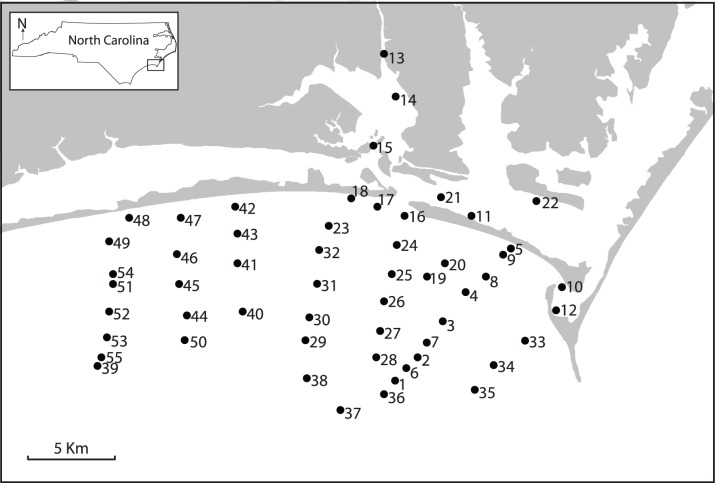
Map of sample locations. Inset box in top left corner shows location of the field area in North Carolina. Each locality (numbered 1–55) was sampled a minimum of three times. In the restricted data analyses, open ocean localities are all localities except 10–15 and 21–22, and the small grid of samples consists of localities 39–55.

Samples were collected in a series of dredges, near the city of Beaufort, North Carolina, U.S.A. (Figure 1). Sampling was completed in four field seasons (June 2011, November 2011, May of 2012, and April 2013) to capture seasonal variation in community composition. Repeat visits to localities served to minimize seasonal variation in sample composition and reduce the magnitude to which richness and relative abundances in the living population may be underestimated. Day et al. [12] conducted similar surveys of the benthic invertebrate fauna repeated in different seasons within a year, and determined that seasonal effects on faunal composition were negligible in the study area.

Dredging was conducted at 55 localities, resulting in a total of 221 dredge samples collected from a variety of habitats, depths, and distances from shore (Table 1). At each locality a minimum of three samples were collected, utilizing the following types of equipment (minimum one sample each): a benthic sled, a dredge basket, and a van Veen grab. The benthic sled was lined with 1 mm wire mesh to ensure representative sampling of smaller species and juveniles, and van Veen samples were wet sieved (1 mm mesh). In the case of benthic sled and basket samples, the entire sample was carefully examined, and all invertebrates identifiable without the aid of a microscope, with the exception of small encrusting species (such as bryozoans and sponges), were counted and identified to the lowest taxonomic level possible (typically species). The resulting live samples consist of multiple higher taxonomic groups (e.g., bivalves, gastropods, arthropods, echinoderms, annelids, etc.). Although surficial dredging of this nature provides limited coverage of the sea floor both in terms of surface area covered, and depth below sediment-water interface, infaunal organisms were adequately represented; 27% of live species surveyed and 36% of individuals were infaunal.

**Table 1:** Sample location data.

Locality	Samples	Richness	Abundance	Depth (m)	GPS
1	3	3	47	19.0	N34° 34.383′ W76° 40.474′
2	3	12	47	18.8	N34° 35.044′ W76° 39.650′
3	6	18	105	18.3	N34° 36.506′ W76° 38.372′
4	6	29	256	16.7	N34° 37.691′ W76° 37.404′
5	3	25	409	11.7	N34° 39.516′ W76° 34.956′
6	3	9	63	18.7	N34° 34.863′ W76° 39.714′
7	3	20	72	18.7	N34° 35.888′ W76° 38.913′
8	6	36	408	15.5	N34° 38.363′ W76° 36.237′
9	5	22	247	8.0	N34° 39.473′ W76° 34.576′
10	3	21	119	8.5	N34° 38.258′ W76° 32.752′
11	6	55	562	9.9	N34° 40.698′ W76° 36.834′
12	4	31	537	8.8	N34° 37.449′ W76° 32.854′
13	6	23	435	5.2	N34° 48.006′ W76° 41.216′
14	8	40	398	5.4	N34° 45.751′ W76° 40.562′
15	8	45	593	5.6	N34° 43.445′ W76° 41.622′
16	8	25	1253	13.6	N34° 40.877′ W76° 39.929′
17	6	12	73	6.7	N34° 41.307′ W76° 41.185′
18	7	18	283	7.9	N34° 41.460′ W76° 42.554′
19	5	30	189	9.6	N34° 40.499′ W76° 38.665′
20	4	17	208	15.5	N34° 39.028′ W76° 38.063′
21	7	16	235	8.3	N34° 41.528′ W76° 38.142′
22	5	29	213	4.9	N34° 41.374′ W76° 33.769′
23	3	22	179	16.0	N34° 40.375′ W76° 43.591′
24	3	31	779	15.0	N34° 39.755′ W76° 40.362′
25	3	21	116	16.3	N34° 38.595′ W76° 40.642′
26	3	16	87	17.0	N34° 37.585′ W76° 40.868′
27	3	13	56	18.0	N34° 36.316′ W76° 41.138′
28	3	15	65	18.3	N34° 35.403′ W76° 41.331′
29	6	14	62	18.0	N34° 35.952′ W76° 44.557′
30	3	14	91	17.5	N34° 36.819′ W76° 44.487′
31	3	18	134	16.0	N34° 38.218′ W76° 44.358′
32	3	22	128	17.0	N34° 39.459′ W76° 44.176′
33	3	19	217	16.0	N34° 35.740′ W76° 34.505′
34	3	9	60	18.0	N34° 34.928′ W76° 35.792′
35	3	9	55	18.0	N34° 34.009′ W76° 36.660′
36	3	8	59	19.0	N34° 33.921′ W76° 40.914′
37	3	7	64	19.0	N34° 33.230′ W76° 43.016′
38	3	13	82	18.7	N34° 34.442′ W76° 44.632′
39	3	8	106	18.3	N34° 34.939′ W76° 54.526′
40	3	15	82	16.9	N34° 37.281′ W76° 47.791′
41	6	21	143	16.8	N34° 38.852′ W76° 48.196′
42	4	21	137	13.3	N34° 41.149′ W76° 48.010′
43	4	19	207	16.0	N34° 40.118′ W76° 48.143′
44	4	33	356	17.2	N34° 36.891′ W76° 50.267′
45	3	20	319	17.0	N34° 38.122′ W76° 50.702′
46	3	22	442	16.0	N34° 39.306′ W76° 50.736′
47	3	21	205	13.0	N34° 40.761′ W76° 50.570′
48	3	16	257	11.3	N34° 40.739′ W76° 53.045′
49	3	20	251	16.0	N34° 40.015′ W76° 54.028′
50	3	15	100	16.0	N34° 35.938′ W76° 50.392′
51	6	20	129	16.3	N34° 38.005′ W76° 53.658′
52	3	12	94	17.0	N34° 37.060′ W76° 54.003′
53	3	12	126	17.0	N34° 36.077′ W76° 54.068′
54	3	12	546	17.0	N34° 34.772′ W76° 54.699′
55	3	12	143	18.0	N34° 35.938′ W76° 50.392′

Number of samples collected at each locality is listed under “Samples”.

Sampling resulted in an abundance matrix consisting of 11248 individuals, 231 species, and 220 samples from 55 localities. Localities with less than 30 individuals, species with less than 10 individuals, and taxa occurring only at a single locality were removed from the data matrix (see below for justification). The resulting matrix, consisting of 9505 individuals (Table S1), 69 species (Table S2), and 49 localities (Table S3), was used as the initial dataset in all subsequent analyses. Data were analyzed using common multivariate ordination techniques: Principal Coordinates Analysis (PCoA or Classical Multidimensional Scaling), Correspondence Analysis (CA), Detrended Correspondence Analysis (DCA), and Non-Metric Multidimensional Scaling (NMDS). The bivariate relationship between community composition and bathymetry was evaluated via a reduced major axis regression of scores from Axis 1 of a given ordination against the appropriate water depth (m) estimates (locality water depths or weighted species occurrence depths). Locality water depth was recorded when sampling localities, using the onboard depth sounder (±0.3 m).

Weighted species occurrence depths were obtained for each species by weighted averaging of a species across all localities in which that species was present. For example, Species A found in two localities only, with Locality X at 10 m including *n* = 5 specimens and Locality Y at 8.5 m including *n* = 100 specimens, would have a preferred water depth calculated as (10*5+8.5*100)/(100+5) = 8.57 m. Note that this approach represents a direct ordination method based on direct measurements of water depth.

Multiple indirect ordination strategies have been developed for community and other compositional data, including four widely used methods. PCoA represents relationships between objects in multidimensional space, and involves translation of dissimilarities between objects into Euclidian distances [34], [35], [36]. Ordinations produced in PCoA, however, can distort ordination gradients [37], especially for sparse data matrices, forcing long gradients into curved patterns (the horseshoe effect). CA involves the repeated averaging of scores, maximizing the correspondence between species and locality scores [38], [39], and can also suffer from curvilinear distortions (the arch effect) and compression of gradient extremes. DCA was developed to counter these distortions [40]. DCA divides the first axis into segments and centering each segment on zero of Axis 2 (detrending) removing any systematic relationship between scores, and shifting the positions of localities along the first ordination axis by rescaling the segments [40]. This straightens out the arch generated by correspondence analysis, and the ends of the gradient tend to be less compressed. This rescaling may not, however be desirable in all cases [37], [41]. NMDS is an iterative technique that optimizes the placement of samples into a low-dimensional space minimizing the mismatch between rank-order of multivariate ecological dissimilarity and Euclidean distances in NMDS space [42], [43], [44], [45]. NMDS is also potentially affected by arch effect and, in addition, this non-eigenvector method does not maximize the variability associated with individual axes [46]. These indirect ordination methods (especially CA, NMDS, and DCA) are widely employed in ecology and paleoecology, but numerous controversies surround their relative effectiveness and validity (e.g., [20], [31], [41], [46]). Analyses were therefore performed using all four types of ordination techniques to both facilitate comparison with other studies of both modern and ancient communities, and to compare their relative performance in the specific case of the data analyzed here.

To evaluate the effect of selective restriction of community data (which is inevitable in the case of paleontological data and, in practice, also affects ecological community analysis), ordinations were performed using all taxa, preservable taxa, and heavily biomineralized mollusks to imitate common types of paleontological data. Preservable taxa were defined herein as species with biomineralized skeletal components, which therefore could potentially be preserved in the fossil record (e.g., arthropods, echinoderms, mollusks). This category excluded organisms with no macroscopic hard-parts, such as polychaetes, sponges, and soft corals. Heavily biomineralized mollusks consisted of species with thick shells, having a relatively high preservation potential. Note that the heavily biomineralized mollusk category restricted the analysis to one higher taxon and also eliminated mollusk species that have thin, fragile shells (e.g., *Anomia simplex*) or lack shells (e.g., nudibranchs).

To investigate the effect of spatial scaling on the relationship between bathymetry and community composition, results were compared for the study area (all localities, area ∼952 km^2^, depth range 4–20 m, and variable salinity), open ocean habitats (all localities excluding 10–15 and 21–22, area ∼578 km^2^, depth range 7–19 m, and relatively invariant salinity), and a small grid of samples along an onshore-offshore gradient (localities 39–55, area ∼180 km^2^, depth range 11–18 m, and relatively invariant salinity). At finer spatio-temporal scales, samples may not ordinate along bathymetric gradients [30], and consequently, the strength of the relationship between bathymetry and community composition in ordination space may be expected to deteriorate.

The above datasets, selectively restricted by taphonomic, taxonomic, and geographic criteria, are subsets of the entire dataset. The ordinations based on reduced datasets may be expected to perform less effectively in regression models owing to loss of information associated with loss of taxa, localities, and specimens, resulting in lower values of R^2^, merely as an artifact of reduced dimensions of the data matrix and smaller numbers of specimens per sample and/or per taxon. Random subsampling without replacement was therefore used to generate replicate random subsets of the total dataset while mimicking dimensions and sample sizes of the restricted datasets analyzed above. NMDS was performed for each randomly resampled dataset, and only NMDS ordinations with a stress of 0.2 or lower were retained. Stress values >0.2 are generally considered poor and potentially uninterpretable [31], [47]. Although such generalizations regarding the interpretability of stress values are arguably oversimplified as stress values vary based on the number of samples and species [47], [48], the 0.2 cutoff value is still relatively lenient, allowing us to include many more outcomes in resampling simulations. Outcomes of individual simulation runs are thus more variable, producing more conservative estimates of standard errors. In addition, NMDS analyses performed in three dimensions (which inevitably notably reduced stress values) produced ordinations consistent with their two-dimensional counterparts (only the latter results are reported here). For all NMDS runs with stress <0.2, R^2^ values were computed for Axis 1 scores and the independent depth estimates. 1000 iterations were run for each subset. The distribution of the resampled R^2^ values were compared with the actual values observed for the entire dataset and for the actual restricted datasets. Separate simulations were performed for each of the four restricted dataset (preservable, robust mollusks, open ocean, and onshore-offshore gradient). The offset between the mean R^2^ values obtained in simulation and the R^2^ value for the total dataset provide an estimate of the bias due solely to the effect of data restriction. Note that if the R^2^ value for a given restricted dataset approximates the mean R^2^ value in the corresponding simulation, the poorer performance of the restricted dataset can be attributed to sampling information loss rather than lower informative value of the specific non-random subset of data targeted in a given restricted analyses. Conversely, departures from the model prediction would suggest that the restricted dataset is less (or more) informative than would be expected for a random subset derived from the entire dataset.

**Figure 2:**
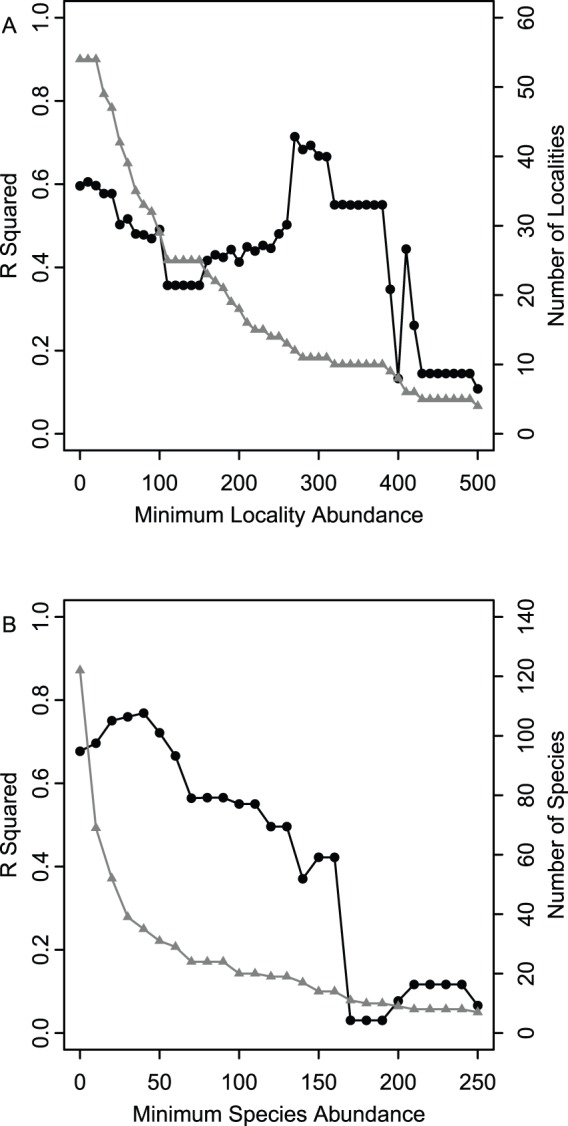
Locality and species abundance filtering. (A) Dashed grey line indicates number of localities retained at a given minimum acceptable sample size. Solid black line indicates adjusted R^2^ values from regression of Axis 1 scores with locality depths. (B) Grey line with triangles indicates number of species retained at a given minimum acceptable sample size (i.e., a minimum acceptable number of specimens per species). Solid black line with circles indicates adjusted R^2^ values from regression of Axis 1 scores with weighted species occurrence depths. In both figures, each R^2^ value represents a new ordination. Note that a minimum species abundance of 10 and minimum locality abundance of 30 was used for all analyses, which allowed us to retain the bulk of the data and R^2^ consistent with values observed for larger sample sizes.

Minimum acceptable sample size per locality was determined by performing NMDS, and correlating Axis 1 scores with species and locality depths at 10 specimen increments. Iterative removal of localities demonstrated that R^2^ values were relatively stable even when localities with n<30 were included, and only became volatile above the threshold value of n = 280 (Figure 2A). In contrast, for species, R^2^ values were relatively stable regardless of the threshold. Consequently, a relatively small threshold value of n = 10 specimens per species was used, which allowed the retention of the majority of species and a substantial fraction of localities in the final analysis.

All ordinations and analyses were performed using R (version 2.15.1; Tables S4-S7). PCoA (Bray-Curtis distance) and DCA (Chi-square distance) were performed using the “Vegan” package [49]. The default setting of 26 segments was used in all DCA analyses. NMDS (Bray-Curtis distance) and CA (Chi-squared distance) were performed using the “MASS” package [50]. NMDS was performed using two and three dimensions (see above).

Reduced major axis regression (RMA), also known as Standard Major Axis Regression, was used to develop linear models relating water depth and Axis 1 ordination scores. This method is particularly applicable here because the compared variables (ordination scores and water depth) are of intrinsically different types and thus require standardization prior to analysis (e.g., [46]). Furthermore, as substantial errors potentially affect both compared variables, a Type II regression model is more appropriate (e.g., [51]). RMA models were based on ordination scores regressed against depth values (either locality depth or weighted species occurrence depths).

All R^2^ values reported herein are the adjusted R^2^ values. Whereas adjusted R^2^ is most commonly applied to multiple regression problems (to compensate for adding additional effects to the model), it is also appropriate for simple linear regression when data represent a sample, rather than exhaustive data for entire statistical populations [46]. The adjusted R^2^ is always lower than unadjusted R^2^ and provides a more conservative estimate of amount of variance accounted for by the independent effect variable. Because our samples are generally large and all models are 1-parameter models, the differences between the adjusted R^2^ and non-adjusted R^2^ are trivial (often non-observable when R^2^ value is rounded to 2 decimal places). Pearson’s correlation coefficient (*r*) and 95% confidence intervals were calculated for each data subset using both standard parametric approximations and bootstrap resampling. Each bootstrap estimate was based on 1000 replicate samples obtained by sampling pairs of observations with replacement.

## Results

**Figure 3:**
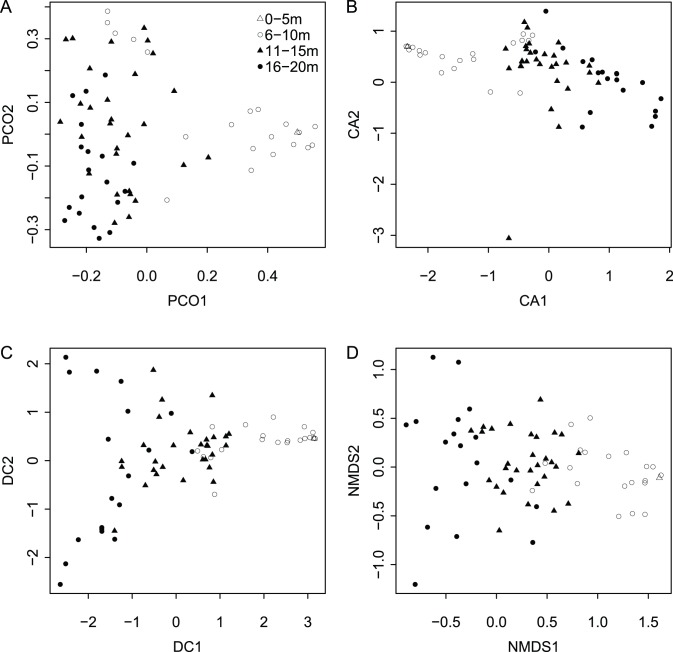
Ordination species score plots for all four ordinations types. (A) PCoA (Axis 1 = 18%, Axis 2 = 12%), (B) CA (principal inertias CA1 = 51%, CA2 = 49%), (C) DCA, (D) NMDS (stress 0.19). Symbols in (A), top right, denote depth ranges.

**Figure 4:**
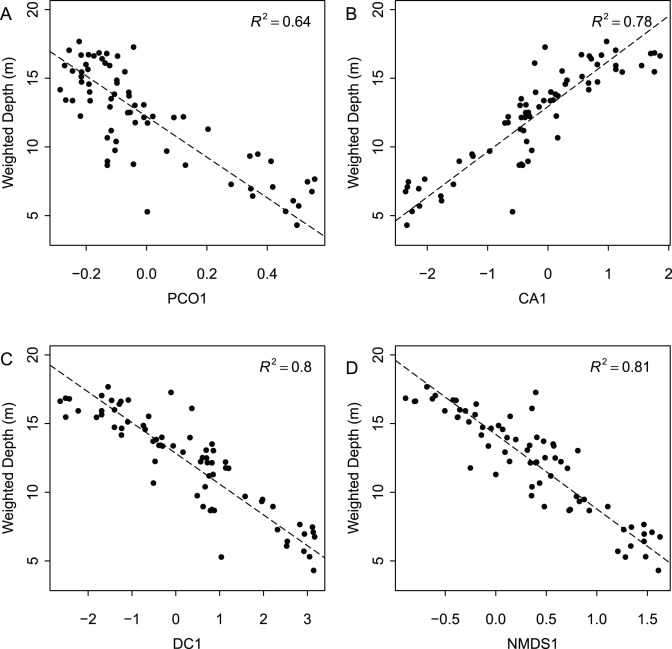
Reduced major axis regression of weighted species depth estimates and Axis 1 species scores for four types of ordinations. (A) Principal Coordinates Analysis, (B) Correspondence Analysis, (C) Detrended Correspondence Analysis, (D) Non-Metric Multidimensional Scaling.

**Figure 5:**
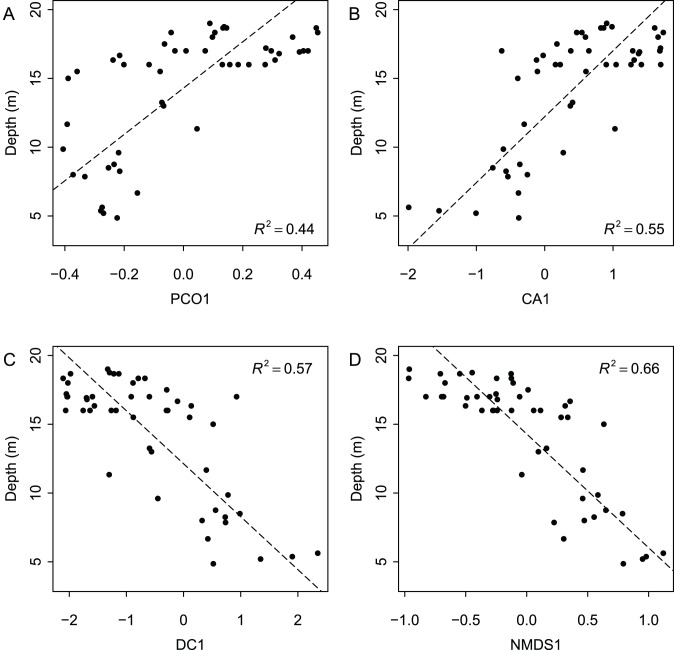
Reduced major axis regression of locality depth and Axis 1 locality scores for four types of ordinations. (A) Principal Coordinates Analysis, (B) Correspondence Analysis, (C) Detrended Correspondence Analysis, (D) Non-Metric Multidimensional Scaling.

**Figure 6:**
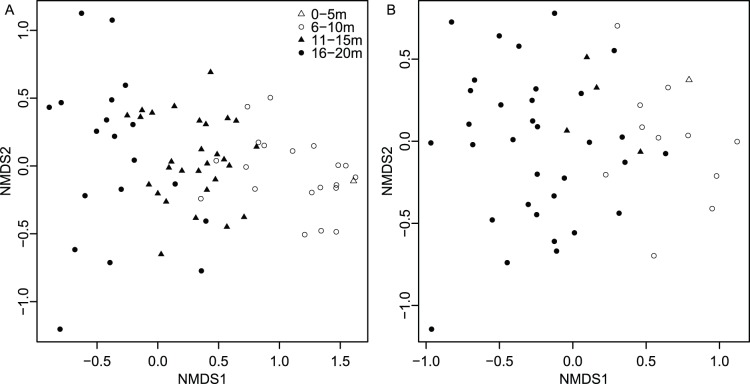
NMDS plots of locality and species scores. (A) plot by species, (B) plot by localities. Symbols denote depth ranges of species (A) or localities (B).

When species ordination scores are plotted by depth range (Figure 3), species separate by depth along the first axis for all four ordination techniques. Axis 1 scores are a significant predictor of weighted species occurrence depths for all four ordination techniques (Figure 4). Adjusted R^2^ values are comparable across ordination methods, with Axes 1 yielding the highest values for NMDS and DCA (0.81 and 0.80 respectively), and lowest for PCoA (0.64). Although regressions between Axis 1 scores and locality depth are also all significant (*p*<<0.0001), R^2^ values are lower (Figure 5). Again, NMDS yielded the highest (0.66) and PCoA the lowest (0.44) R^2^ value. As all four ordination techniques produced similar regression results, all subsequent analyses focused on the NMDS results. The depth gradient is clearly apparent in the NMDS ordination plot (Figure 6), with species and locality depths increasing to the left on Axis 1 (stress = 0.2). Axis 2 species and samples do not align or group according to habitat type or distance from the coastline. Whereas scores for the first axes are highly consistent across different ordination types with absolute value of *r* exceed 0.7 for all comparisons (Table 2), the scores for the second axes are poorly correlated, with most absolute values of *r* below 0.2 (Table 2).

**Table 2:** Axis score correlation coefficients.

Method	*r* (L)	*r* (Sp)
CA1 vs. DC1	−1.00*	−1.00*
CA1 vs. MDS1	−0.81*	−0.91*
CA1 vs. PCO1	0.91*	−0.84*
PCO1 vs. MDS1	−0.88*	0.74*
DC1 vs. MDS1	0.81*	0.91*
DC1 vs. PCO1	−0.90*	0.84*
CA2 vs. DC2	0.09	0.11
CA2 vs. MDS2	−0.06	−0.09
CA2 vs. PCO2	0.21	0.54*
PCO2 vs. MDS2	0.56*	0.08
DC2 vs. MDS2	−0.19	−0.09
DC2 vs. PCO2	−0.48*	0.11

Spearman’s Rank correlation coefficients (*r*) of Axis 1 and Axis 2 locality (L) and species (Sp) scores for each ordination method.

*Denotes significant correlations (α = 0.05).

**Figure 7:**
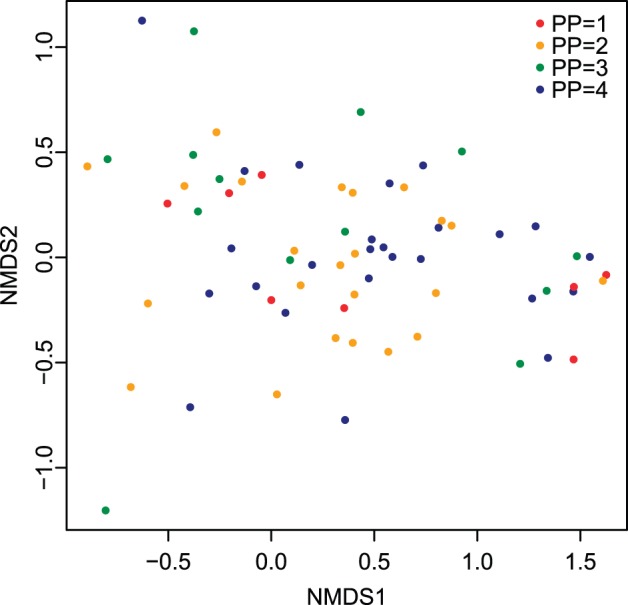
NMDS plot of species scores and preservation potential. Symbols denote relative preservation potential (4 = heavily biomineralized, 3 = fragile but skeletonized hard parts, 2 = some hard parts such as chitin, 1 = all soft parts). Note that species with various ranks of preservation potentials are distributed along the entire range of Axis 1.

**Figure 8:**
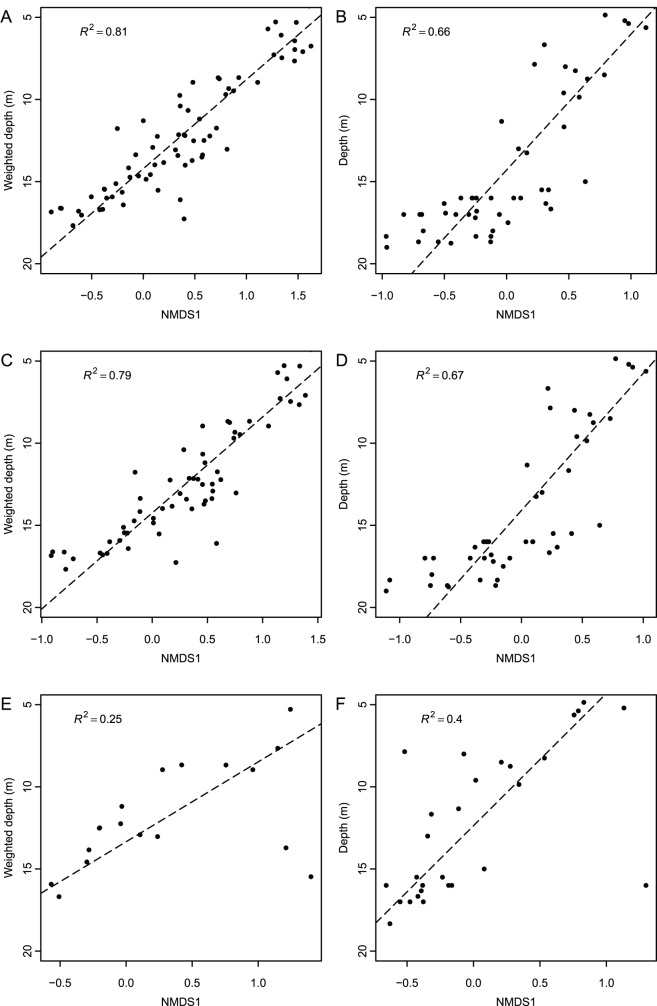
Effects of taphonomic biases and taxonomic scaling. Reduced major axis regressions between Axis 1 scores and depth for: (A–B) all taxa, (C–D) preservable taxa (stress 0.2), and (E–F) heavily biomineralized mollusks (stress 0.2). Plots on the left utilize species scores, plots on the right use locality scores. Note the presence of two notable outliers in 8E (bottom right quadrant, bivalves *Spisula solidissima* and *Ensis directus*), and one in 8F (bottom right quadrant, locality 49), without which the relationship between species or sample depth and Axis 1 scores would greatly improve.

When data are restricted to preservable taxa only (46 localities, 61 species, 7545 individuals, Table S8; Figure 7), the regression for species scores is still significant, although not as strong (Figure 8 C; R^2^ = 0.79, *p*<<0.0001). When only heavily biomineralized mollusks are retained (28 localities, 18 species, 3042 individuals, Table S9) the relationship weakens considerably (Figure 8 E; R^2^ = 0.25, *p* = 0.003). Locality scores performed similarly; when preservable taxa are retained the results are nearly identical to those obtained for all taxa (Figure 8 D; R^2^ = 0.67, *p*<<0.0001), but the association between depth and locality scores weakens considerably when only heavily biomineralized mollusks are retained (Figure 8 F; R^2^ = 0.40, *p* = 0.04). It should be noted that organisms which vary in preservational potential (i.e., heavily biomineralized species, species with fragile but skeletonized hard parts, species with some hard parts such as chitin, and species with soft tissues only) are present along the entire length of Axis 1 (Figure 7). Thus, the differences observed across the three compared datasets (all taxa, preservable taxa, and heavily biomineralized mollusks) are unlikely to represent an artifact of a non-random clustering of organisms with high (versus low) preservation potential along the first ordination axis.

**Figure 9:**
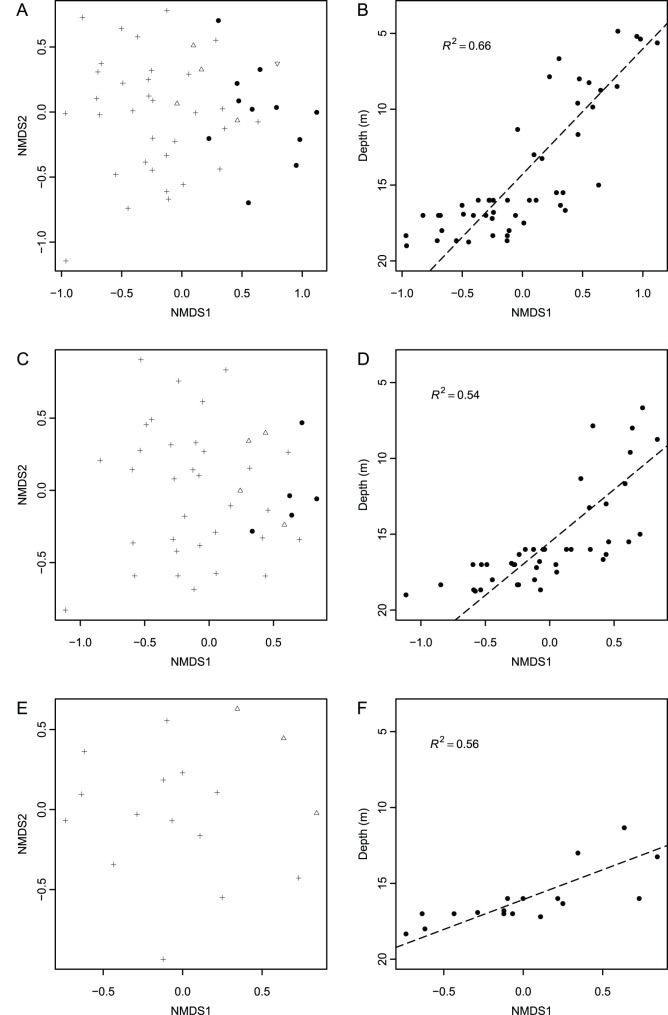
Effects of geographic scaling. NMDS locality score plots and corresponding reduced major axis regressions between Axis 1 locality scores and depth for: (A–B) the entire study area (stress 0.2), (C–D) open ocean localities only (stress 0.2), (E–F) a small onshore-offshore gradient (stress 0.17). Symbols in NMDS plots denote depth ranges (reversed triangle = 0–5 m, filled circle = 6–10 m, inverted triangle = 11–15 m, cross = 16–20 m).

NMDS ordinations performed at successively smaller spatial scales yielded qualitatively consistent results. The dimensions of the resultant restricted datasets are as follows: open ocean localities consists of 42 localities, 52 species, 7120 individuals (Table S10), and the onshore-offshore gradient consists of 17 localities, 50 species, 2830 individuals (Table S11). Localities separate by depth along the first axis for all localities (Figure 9 A), open ocean localities (Figure 9 C), and for the onshore-offshore gradient (Figure 9 E). Axis 1 scores are a significant predictor of locality depths for open ocean localities (Figure 9 D; R^2^ = 0.56, *p*<<0.0001), and the onshore-offshore gradient (Figure 9 F; R^2^ = 0.54, *p*<0.001), although the strength of the relationship is weaker, in both cases, when compared to the results for all localities (Figure 9 B; R^2^ = 0.66, *p*<<0.0001).

**Figure 10:**
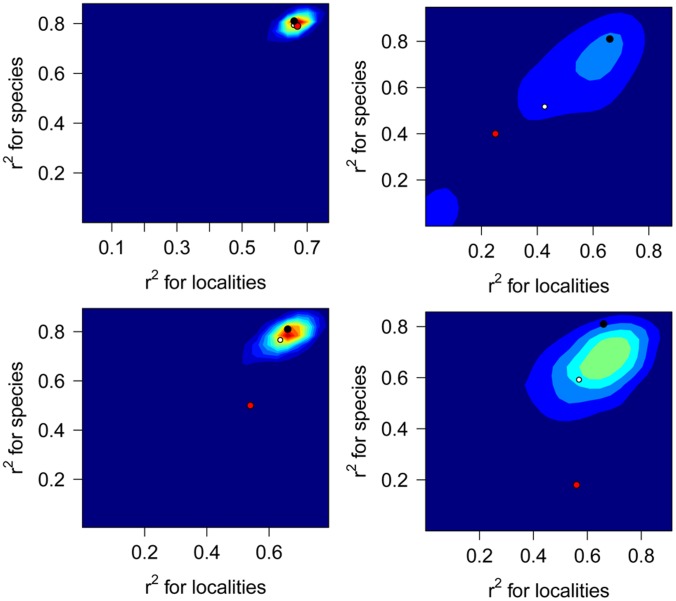
Distributions of r^2^ values resulting from random resampling. (A) Preservable Taxa, (B) Heavily Biomineralized Mollusks, (C) Ocean localities, and (D) Small Grid. White dot denotes mean of simulations, red dot denotes actual observed value, and black dot denotes value for complete data set. Color contours within plots denote density from low (dark blue) to high (red).

When compared to the observed r^2^ value for the total dataset, the mean R^2^ values estimated by random subsampling are notably lower for heavily biomineralized mollusks, open ocean localities, and the onshore-offshore gradient (Figure 10), but comparable for preservable taxa. Note that the simulations for preservable taxa and open ocean localities result in the removal of a small number of taxa and localities from the dataset. For three out of the four restricted datasets (robust mollusks, open ocean localities, and the onshore-offshore gradient), the observed R^2^ values for the subsets of data are lower than the mean R^2^ values produced by random subsampling. Simulated R^2^ values for heavily biomineralized mollusks and the onshore-offshore gradient, are widely dispersed. Pearson’s correlation coefficients are all negative and statistically significant (Table 3). The absolute values of coefficients are high (mean = −0.73) and 95% confidence intervals are relatively narrow, with the exception of the two datasets with smaller sample sizes (robust mollusks with 18 species and 28 localities, and the onshore-offshore gradient with 50 species and 17 localities).

**Table 3:** Confidence intervals for correlation coefficients.

Species Scores	*n*	*r*	*p*	Lower CI	Upper CI	Lower CI(bootstrap)	Upper CI(bootstrap)
Entire Dataset	69	−0.90	<<0.0001	−0.94	−0.85	−0.94	−0.85
Preservable	61	−0.89	<<0.0001	−0.93	−0.83	−0.94	−0.83
Robust Mollusks	18	−0.54	0.02	−0.80	−0.10	−0.94	−0.03
Open Ocean	52	−0.72	<<0.0001	−0.83	−0.55	−0.83	−0.57
Grid Transect	50	−0.45	0.001	−0.65	−0.20	−0.64	−0.21
**Locality Scores**							
Entire Dataset	49	−0.82	<<0.0001	−0.89	−0.70	−0.89	−0.72
Preservable	46	−0.82	<0.0001	−0.90	−0.70	−0.90	−0.73
Robust Mollusks	28	−0.65	0.002	−0.83	−0.37	−0.93	−0.28
Open Ocean	42	−0.74	<<0.0001	−0.85	−0.57	−0.84	−0.64
Grid Transect	17	−0.77	0.0003	−0.91	−0.46	−0.91	−0.59

Pearson correlation coefficients, parametric *p*-values, and 95% confidence intervals (CI) are shown for the entire dataset, and each data subset.

## Discussion

A strong relationship between bathymetry and faunal composition of marine benthic communities was observed along the studied onshore-offshore gradient suggesting that environmental factors associated with increasing water depth were the primary factor controlling faunal composition of the marine benthos in the study area, despite substantial habitat heterogeneity. Thus, although multiple gradients likely affected community composition in this region, water depth appears to have been a strong correlative of primary processes that control community composition. Altering the minimum acceptable sample size required for retention of localities and species did not obscure the observed relationships between ordination outcomes and the bathymetry, which appeared relatively strong even when rare taxa and localities with low abundance were included. Regressions between the known depth and Axis 1 ordination scores indicated that all four ordination techniques identified bathymetry (and its implied environmental correlatives) as the greatest source of variation in the data, with NMDS scores yielding the highest R^2^ values. NMDS produced ordinations highly consistent with other methods despite relatively high stress values (cutoff value of 0.2). These results are consistent with previous studies concluding that the results of DCA and NMDS are often comparable [20]. The second axis could not be interpreted within the context of the available environmental information. Moreover, the ordinations of samples and species are highly inconsistent across different ordination techniques suggesting strong and variable distortions along second axes.

When only preservable taxa were retained in the analysis, the bathymetric signal was still detectable, although somewhat weaker relative to ordinations using the entire community. Preservable taxa therefore served as a reasonable proxy for all taxa. Because these taxa could potentially be preserved in the fossil record, the results suggest that indirect ordinations of fossil communities may provide viable proxies for bathymetric gradients in cases when such gradients were ecologically important. Although the use of restricted datasets limited to preservable species should provide a proxy for fossil communities, such data do not incorporate the potential effects of substantial time-averaging that affects fossil samples [52], [53], [54], [55], [56], [57]. However, time-averaging tends to average seasonal variations in faunal compositions and enhance alpha diversity [58], [59], [60], while exerting only small loss of information on beta diversity [56], [58]. Consequently, it is likely that time averaged samples would perform adequately in cases when the preservable part of the community offers an informative proxy for environmental gradients. Finally, the dataset restricted to robust mollusks was less effective in detecting the bathymetric gradient suggesting that, for our study area, the restriction of data to robust mollusks would hamper paleontological analyses aimed at detecting environmental gradients. While the poor performance of mollusks cannot be readily evaluated from the existing data, the underperformance of robust mollusks may be due to lower diversity/high evenness (only 13 species total) and, possibly, broad bathymetric distributions of some of the most abundant species (e.g., *Mulinia lateralis* is known to occur in depths up to 119 m, *Spisula solidissima* up to 276 m, and *Chione cancellata* up to 108 m).

When analyses are restricted spatially to a single onshore-offshore gradient reducing the geographic area from ∼952 to 180 km^2^ and the depth range from 16 to 7 m, the bathymetric gradient is poorly manifested in ordination patterns. This outcome is not inconsistent with the hypothesis proposed by Redman et al. [30], that multivariate ordinations at fine spatial scales may be unable to detect bathymetric gradients. At finer spatial scales, water depth may no longer be the strongest control on the distribution of taxa, and factors that were secondary at larger spatial scales may become dominant (e.g., life mode and grain size).

Indirect ordination methods applied to marine benthic communities spanning multiple habitats along a depth gradient appear suitable for detecting environmental gradients even when restricted to non-random subsets of organisms that can be preserved in the fossil record. The results reported here reinforce paleontological studies that successfully employed such analytical strategies in the fossil record. However, restricting data to one group of organisms or smaller spatial scales, where water depth may no longer be the primary control, may reduce the effectiveness of multivariate ordination methods to delineate bathymetric gradients even when strong gradients can be detected when multiple groups of benthic organisms are analyzed simultaneously.

## Supporting Information

Table S1 **Filtered dataset used for all initial analyses.** Species by locality abundance matrix, rows are localities and columns are species.
(CSV)

Table S2 **Species list.** Species number listed in column one, weighted species depth (m) in column two, preservation potential in column 3 (1 being no hard parts, 3 being robust, see text for full description of categories), and range of depths in column 4 (1 = 0–4.9 m, 2 = 5–9.9 m, 3 = 10–14.9 m, 4 = 15–20 m). To be used with R script.
(CSV)

Table S3 **Locality list.** Locality number listed in column one, depth (m) in column two, general habitat type in column 3 (1 = harbor, 2 = back-sound, 3 = 0–3 miles offshore, 4 = 6–4 miles offshore, 5 = river), anthropogenic effects in column 4 (1 = least disturbed, 2 = moderately impacted, 3 = highly impacted), depth range (1 = 0–4.9 m, 2 = 5–9.9 m, 3 = 10–14.9 m, 4 = 15–20 m). To be used with R script.
(CSV)

Table S4 **R Script.** Script to perform all analyses, associated data files must be downloaded.
(R) Click here for additional data file.

Table S5 **Weighted species depths.**
(CSV)

Table S6 **Locality depths.**
(CSV)

Table S7 **Filtered dataset used for all initial analyses for resampling analysis.** Species by locality abundance matrix, rows are localities and columns are species, row and column headings have been removed.
(CSV)

Table S8 **Filtered dataset with preservable taxa.** Species by locality abundance matrix, rows are localities and columns are species. Taxa with no macroscopic hard parts have been removed.
(CSV)

Table S9 **Filtered dataset with robust mollusks.** Species by locality abundance matrix, rows are localities and columns are species. Only robust mollusks have been retained.
(CSV)

Table S10 **Filtered dataset with open ocean habits.** Species by locality abundance matrix, rows are localities and columns are species. Only open marine localities have been retained.
(CSV)

Table S11 **Filtered dataset with an onshore-offshore transect.** Species by locality abundance matrix, rows are localities and columns are species. Only localities form a small set of transects have been retained.
(CSV)
